# The Specific Carbohydrate Diet: Obstacle and Opportunities

**DOI:** 10.1097/PG9.0000000000000240

**Published:** 2022-07-29

**Authors:** Paul A. Rufo

**Affiliations:** From the Center for Inflammatory Bowel Disease, Boston Children’s Hospital, Boston, MA.

The inflammatory bowel diseases, including ulcerative colitis and Crohn’s Disease, are chronic intestinal inflammatory disorders, likely resulting from a confluence of genetic vulnerabilities and environmental exposures. Existing therapies center on the use of broad-spectrum anti-inflammatory and immunosuppressive medications. Although newer classes of biologic therapies have introduced some mechanistic specificity, most patients and parents remain concerned about the potential effects of these agents when used in the long term.

Existing data have implicated the intestinal microbiome in the pathogenesis of inflammatory bowel disease (1). Although our understanding is incomplete, it is apparent that certain bacteria or bacterial compositions can impose a dysbiosis that contributes to the development of an inflammatory intestinal milieu. The most effective long-term approach to impact the intestinal microbiome is sustained dietary interventions. Although isolated dietary restriction or supplementation has generally been ineffective, the application of the specific carbohydrate diet (SCD) can affect the constituents of the microbiome (2). Adherence to SCD can favorably impact clinical symptoms and quality of life in patients with inflammatory bowel disease (3). More recent studies have demonstrated endoscopic improvement in response to this diet approach (4).

Suskind et al (5) report the obstacles associated with initiating and maintaining a specific carbohydrate diet (SCD). The authors interviewed parents of 10 patients who had either tried or opted not to try the inclusion of SCD in their child’s IBD management. Two patients opted not to try the SCD. One patient had concomitant dietary restrictions that would have made any further elimination untenable, and another patient had severe disease mandating a more expedient pharmacologic approach. Of those patients that initiated the SCD, four discontinued the diet. Three did so because of a lack of perceived effect. However, the study was not designed nor powered to comment on the clinical impact of SCD on patients. The fourth patient developed a *Clostridium difficile* infection requiring antibiotic therapy and opted not to restart the study.

This article is important because it is among the first to systematically report the difficulties in pursuing this type of dietary intervention. They collected data from semistructured interviews and used an inductive coding regimen to develop thematic categories. Even among the relatively small sample size included in this study, it became clear that the biggest obstacles to pursuing a SCD included cost, time spent preparing special foods/meals, and the psychosocial impact of the diet on the patients’ quality of life.

While the article did not include race or ethnicity data, the authors proposed several factors that have the potential to impact disproportionately less financially resourced and more geographically isolated populations. The less processed foods that are the mainstay of the SCD are typically more expensive per unit than highly processed foods. In addition, the availability and selection of less processed foods are limited in the smaller community markets and convenience stores found in urban centers. This means that patients living in urban centers, who likely have less secure transportation, must bear the added imputed cost of extra time and expenditure when challenged with adhering to the SCD.

The authors propose some potential solutions to address identified barriers to adherence to SCD. FDA designated labeling, analogous to what is afforded to those adhering to gluten-free diets, would make it easier for parents shopping for SCD foods. Designations of this type would also facilitate the provision of SCD-restricted alternatives in school lunches. The ability to access Flexible Spending monies, which are already available to those shopping gluten free, would help offset the added cost of SCD compliant foods. However, these accounts are either unavailable to those working in entry-level, hourly, or per diem jobs or irrelevant to those living paycheck to paycheck.

Studies of this type are critical as investigators and clinicians move forward to identify nonpharmacologic and more holistic approaches to managing chronic diseases, including IBD. Investigators, insurers, and legislators will have to consider the cost/benefit analysis, both in terms of dollars and quality of life that accompanies any recommendations concerning lifestyle and dietary recommendations. It is only by gaining a better understanding of the real or perceived obstacles imparted by these interventions that effective initiatives can be developed.
